# GYY4137 and Sodium Hydrogen Sulfide Relaxations Are Inhibited by L-Cysteine and K_V_7 Channel Blockers in Rat Small Mesenteric Arteries

**DOI:** 10.3389/fphar.2021.613989

**Published:** 2021-03-26

**Authors:** Silvijus Abramavicius, Asbjørn G. Petersen, Nirthika S. Renaltan, Judit Prat-Duran, Roberta Torregrossa, Edgaras Stankevicius, Matthew Whiteman, Ulf Simonsen

**Affiliations:** ^1^ Department of Biomedicine, Pulmonary and Cardiovascular Pharmacology, Aarhus University, Aarhus, Denmark; ^2^ Institute of Physiology and Pharmacology, Lithuanian University of Health Sciences, Kaunas, Lithuania; ^3^ Medical School, University of Exeter, Exeter, United Kingdom; ^4^ Institute of Cardiology, Lithuanian University of Health Sciences, Kaunas, Lithuania

**Keywords:** GYY4137, sodium sulfide, hydrogen sulfide, potassium channels, small mesenteric arteries

## Abstract

Donors of H_2_S may be beneficial in treating cardiovascular diseases where the plasma levels of H_2_S are decreased. Therefore, we investigated the mechanisms involved in relaxation of small arteries induced by GYY4137 [(4-methoxyphenyl)-morpholin-4-yl-sulfanylidene-sulfido-λ5-phosphane;morpholin-4-ium], which is considered a slow-releasing H_2_S donor. Sulfides were measured by use of 5,5′-dithiobis-(2-nitro benzoic acid), and small rat mesenteric arteries with internal diameters of 200–250 µm were mounted in microvascular myographs for isometric tension recordings. GYY4137 produced similar low levels of sulfides in the absence and the presence of arteries. In U46619-contracted small mesenteric arteries, GYY4137 (10^−6^–10^–3^ M) induced concentration-dependent relaxations, while a synthetic, sulfur-free, GYY4137 did not change the vascular tone. L-cysteine (10^−6^–10^–3^ M) induced only small relaxations reaching 24 ± 6% at 10^–3^ M. Premixing L-cysteine (10^–3^ M) with Na_2_S and GYY4137 decreased Na_2_S relaxation and abolished GYY4137 relaxation, an effect prevented by an nitric oxide (NO) synthase inhibitor, L-NAME (N^ω^-nitro-L-arginine methyl ester). In arteries without endothelium or in the presence of L-NAME, relaxation curves for GYY4137 were rightward shifted. High extracellular K^+^ concentrations decreased Na_2_S and abolished GYY4137 relaxation suggesting potassium channel-independent mechanisms are also involved Na_2_S relaxation while potassium channel activation is pivotal for GYY4137 relaxation in small arteries. Blockers of large-conductance calcium-activated (BK_Ca_) and voltage-gated type 7 (K_V_7) potassium channels also inhibited GYY4137 relaxations. The present findings suggest that L-cysteine by reaction with Na_2_S and GYY4137 and formation of sulfides, inhibits relaxations by these compounds. The low rate of release of H_2_S species from GYY4137 is reflected by the different sensitivity of these relaxations towards high K^+^ concentration and potassium channel blockers compared with Na_2_S. The perspective is that the rate of release of sulfides plays an important for the effects of H_2_S salt vs. donors in small arteries, and hence for a beneficial effect of GYY4137 for treatment of cardiovascular disease.

## Introduction

Hydrogen sulfide (H_2_S) is considered an essential signaling molecule in the cardiovascular and nervous systems (Szabó, 2007; Wallace et al., 2018) and a variety of pathophysiological changes including cancer, glycometabolic disorders, diabetes, sepsis, and human malignt hyperthermia are associated with altered endogenous levels of H_2_S (Szabó, 2007; Szabo and Papapetropoulos, 2017; Vellecco et al., 2020). In the cardiovascular system, endogenous H_2_S can lead to both vasodilatation and vasoconstriction (Li et al., 2015; Hedegaard et al., 2016; Gheibi et al., 2018).

Several mechanisms mediate vasodilatation induced by addition of exogenous H_2_S salts, including lowering of smooth muscle cells calcium by activation of K channels (Skovgaard et al., 2011; Hedegaard et al., 2016), enhancement of nitric oxide (NO) signaling (Szabo, 2017), and changes in intracellular pH by inhibition of an acid-sensitive Cl_2_/HCO_3_-exchanger (Lee et al., 2006; Esechie et al., 2008; Malekova et al., 2009; Perniss et al., 2017). The opening of potassium channels leads to hyperpolarization and smooth muscle relaxation. Different types of K channels are involved in H_2_S vasodilatation, including in large arteries ATP-sensitive K channels (K_ATP_) (Zhao and Wang, 2002; Kubo et al., 2007; Webb et al., 2008; Martelli et al., 2013a), voltage-gated K channels (K_V_7, KCNQ) (Martelli et al., 2013a; Hedegaard et al., 2014), and 4-aminopyridine-sensitive voltage-gated potassium channels (Cheang et al., 2010). In resistance arteries, H_2_S vasodilatation involves K_ATP_ channels (Tang et al., 2005), large-conductance calcium-dependent potassium channels (BK_Ca_) (Jackson-Weaver et al., 2011; Jackson-Weaver et al., 2013), and K_V_7 channels (Schleifenbaum et al., 2010; Hedegaard et al., 2016), but also potassium channel-independent vasodilatation (Hedegaard et al., 2016).

In a variety of human diseases, e.g., hypertension and atherosclerotic disease, the plasma levels of H_2_S are decreased (Wang, 2012). Several series of H_2_S donors have recently been developed to substitute for the decreased H_2_S levels (Feng et al., 2015; Steiger et al., 2017; Szabo and Papapetropoulos, 2017). The H_2_S donating compounds compromises of two major groups: the inorganic salts NaHS and Na_2_S, which are rapid H2S releasers, and compounds associated with a slow release of H_2_S, e.g., diallyl disulfide and GYY4137 (4-methoxyphenyl)-morpholin-4-yl-sulfanylidene-sulfido-λ5-phosphane;morpholin-4-ium) (Li et al., 2008; Martelli et al., 2013b) and AP39, AP123, and AP67 (Whiteman et al., 2006). NaSH and Na_2_S produce an instant pH-dependent dissociation to H_2_S and at high concentrations, e.g., 1 mM, induce vasorelaxation (Zhao et al., 2001; Li et al., 2008), while 100 µM GYY4137 associated with release of <1 µM H_2_S is associated with relaxation (Hedegaard et al., 2016).

GYY4137 exhibits vasorelaxant, hypotensive, anti-inflammatory, and anti-cancer activity effects (Li et al., 2008; Martelli et al., 2013a; Lee et al., 2011; Liu et al., 2013), and it is considered as a slow-releasing H_2_S donor (Li et al., 2008; Feng et al., 2015). Different mechanisms have been reported to be involved in the release of sulfide species from GYY4137, including conversion by cystathionine γ-lyase (CSE) (Chitnis et al., 2013) and interaction with cysteine (Martelli et al., 2013b). In a recent study where changes in H_2_S gas were detected with microelectrodes, we observed that GYY4137 induced full relaxation of small mesenteric arteries without producing detectable changes in amperometric currents (Hedegaard et al., 2016). Hence, it is unclear whether H_2_S gas contributing to the pharmacodynamic effects of GYY4137 is below detection level or whether GYY4137 induces vasodilatation by mechanisms independent of H_2_S gas e.g., commercial GYY4137 has dichlormethane leading to formation of carbon monoxide (CO) (Alexander et al., 2015).

To examine whether sulfides are involved in GYY4137 relaxations, measurements of sulfides were conducted and compared to a hydrolyzed version of GYY4137 (Alexander et al., 2015). Na_2_S was chosen for comparison as its vasodilating effects previously have been associated with increases in H_2_S gas (Hedegaard et al., 2016). To investigate whether CSE or L-cysteine contribute to the release of H_2_S from GYY4137, the effect of an inhibitor of CSE and L-cysteine were examined on GYY4137 relaxation. Release of H_2_S from Na_2_S and GYY4137 appear to have different kinetics and that may change the involvement of potassium channels, and therefore relaxations induced by the two compounds were investigated in the presence of blockers of potassium channels. Small mesenteric arteries contribute significantly to vascular resistance and blood pressure in intact animals (Fenger-Gron et al., 1995), and therefore the vasodilatation studies were performed in rat small mesenteric arteries.

## Methods and Materials

### Animals and Preparation of Samples

The investigation was carried out in accordance to the Guide for the Care and the Use of Laboratory Animals published by the United States National Institutes of Health (NIH Publication No. 85–23, revised 1996) and followed the ARRIVE guidelines (McGrath and Lilley, 2015). Adult male Wistar rats (12–14 weeks) were killed by decapitation and subsequent exsanguination. The protocol was approved by the Danish Animal Experiments Inspectorate (permission 2014-15-2934-0159).

### Chemicals and Materials

The following drugs were used: noradrenaline, acetylcholine (ACh), L-NAME (N^ω^-nitro-L-arginine methyl ester), Na_2_S, XE991 [10,10-bis(4-pyridinylmethyl)-9(10H)-anthracenone dihydrochloride], glibenclamide from Sigma-Aldrich (St. Louis, MO), 5,5′-dithiobis-(2-nitro benzoic acid) (DTNB) (Sigma), D,L-propargylglycine (PPG; an irreversible inhibitor of the enzyme cystathionine γ-lyase (CSE), an H_2_S synthase inhibitor), 1,4-Dithiothreitol (DTT). GYY4137 ((4-methoxyphenyl)-morpholin-4-yl-sulfanyl-sulfanylidene- λ5-phosphane sodium salt) was synthesized, as described previously by us (Alexander et al., 2015). Fresh Na_2_S solution was prepared every day. To neutralize pH of the solution, hydrochloric acid was added until a pH of 7.35–7.45 was obtained. The composition of the physiologic salt solution (PSS) was NaCl 119 mM, NaHCO_3_ 25 mM, glucose 5.5 mM, CaCl_2_ 1.6 mM, KH_2_PO_4_ 1.18 mM, MgSO_4_ 1.17 mM, and EDTA 0.027 mM. High potassium solution, KPSS, was PSS with NaCl exchanged for KCl on equimolar basis.

### Measurements of Release of Sulfide Species From GYY4137

Sulfide species (H_2_S and HS^−^) released from GYY4137 were assessed *in vitro* as described previously (Li et al., 2008). In brief, 100 mM phosphate buffer pH 7.40 was incubated with 1.0 or 0.1 mM GYY4137 at 25 or 37°C. A phosphate buffer with pH of 3.01 was also tested as acidic conditions have been shown to promote H_2_S release from GYY4137 (Li et al., 2008; Hedegaard et al., 2016). At appropriate times, 20 µl aliquots were removed and added to 96-well microplates containing 50 µl 1 mM DTNB and 50 µl 1 M HEPES buffer pH 8.0, and absorbance was measured at 412 nm on a plate reader. The concentration of H_2_S formed from GYY4137 was calculated from a standard curve of NaSH (1–500 µM) for each of the respective time points.

### Functional Studies in Small Mesenteric Arteries

Third branch mesenteric arteries were dissected from the mesenteric vascular bed and mounted on 40 µm steel wires in microvascular myographs (Danish Myotechnology, Aarhus, Denmark) for isometric tension recording as previously described (Mulvany and Halpern, 1976). The vessels were equilibrated in oxygenated (5% CO_2_, 20% O_2_, 75% N_2_) PSS at 37°C and for 30 min, and by stretching normalized to a lumen diameter (d_100_) equivalent to 100 mm Hg, after which tension was set to 90% x d_100_ (Mulvany and Halpern, 1976). At this tension, the internal lumen diameters were 200–250 µm. After normalization, the arterial segments were stimulated with KPSS, washed in PSS, and stimulated with noradrenaline (10 µM). Arteries were only included if they developed an active force corresponding to a transmural pressure of 100 mmHg. The PowerLab data system and Chart 5.5 (ADInstruments, Oxfordshire, United Kingdom) was used to record the data. The mechanical responses of the vessel segments were measured as active wall tension (ΔT), which is the changes in force (ΔF) divided by twice the segment length (2L).

### Experimental Protocol

To determine whether the effects of GYY4137 were due to H_2_S released from it, the parent compound was compared with an analog, which is normally produced by a two-step hydrolytic degradation of GYY4137 over weeks, but in this study synthesized as previously described (Alexander et al., 2015).

The biosynthetic enzymes for H_2_S production (CBS and CSE) were reported to be involved in the relaxant effects of GYY4137 in bovine ciliary arteries (Chitnis et al., 2013). Therefore, small mesenteric arteries were incubated with D, L-propargylglycine (PPG, 10 mM), which is an irreversible inhibitor of CSE, and concentration-response curves were constructed for Na_2_S and GYY4137. L-cysteine is considered substrate for formation of endogenous H_2_S (Wang, 2012), and was also reported as a scavenger of HS^−^ giving rise to formation of sulfides (Koike et al., 2017). Concentration-response curves for L-cysteine were constructed in U46619 (0.3 µM)-contracted preparations. To investigate an eventual scavenger effect, we conducted two set of experiments. In a first set of experiments, solutions of cysteine and, respectively, Na_2_S and GYY4137 were pre-mixed in airtight containers and then added to the arteries contracted with U46619 at 0 and 10 h after the mixing. At 10 h the amount of sulfides was also measured using the DTNB assay as described above. In a second series of experiments, small mesenteric arteries were incubated with L-cysteine (10^–3^ M) or the thiol reducing agent, 1,4-dithiothreitol (10^−3^ M), and concentration-response curves were constructed for GYY4137 and Na_2_S. The control and examination of drugs were run in parallel, and only one concentration-response curve was constructed for each vasodilator per animal.

To investigate the role of the endothelium in relaxations induced by Na_2_S and GYY4137, arterial segments with and without endothelium were mounted. The endothelial cells were removed by introducing into to the lumen a human scalp hair and rubbing back and forth several times (Hedegaard et al., 2016). The effectiveness of the procedure was assessed by absence of relaxation to acetylcholine in noradrenaline-contracted arteries, while vessel with endothelium were accepted only if acetylcholine-induced (10^–5^ M) relaxation on noradrenalin-induced (5 × 10^–6^ M) contraction was larger than 50%, and exclusion following these criteria explains unequal group numbers are reported. The preparations were contracted with U46619 (0.3 µM) giving a contraction level corresponding to 50–60% of the contractions induced by 125 mM KPSS, and when the contraction was stable cumulative concentration-response curves were constructed for Na_2_S (10^−6^–10^–3^ M) or GYY4137 (10^−6^–10^–3^ M). Preparations were incubated with the NO synthase inhibitor, L-NAME (300 µM), the preparations were contracted with U46619 (0.1 µM) to obtain contraction similar to controls levels, concentration-response curves were constructed for increasing concentrations of Na_2_S, GYY4137, and acetylcholine.

High concentrations of the NO donor, sodium nitroprusside (SNP) with NaSH yield formation of a nitrosothiol and inhibits NO-induced rat aorta relaxation (Ali et al., 2006; Whiteman et al., 2006). To investigate the interaction with NO, concentration-response curves for SNP (10^−9^–10^–4^ M) were obtained in the absence and presence of GYY4137 or Na_2_S.

The involvement of K channels in Na_2_S and GYY4137 induced relaxation were examined by comparing relaxations in U46619 and high-potassium physiologic saline solution (KPSS)-contracted arteries. To investigate the specific K channels involved, the preparations were incubated for 30 min with a selective blocker of ATP-sensitive K channels, glibenclamide (1 µM) (Mulvany et al., 1990), a selective blocker of large-conductance calcium-activated K channels, iberiotoxin (100 nM) (Giangiacomo et al., 1992), and a blocker of voltage-gated K_V_7 channels, XE991 (10 µM) (Yeung et al., 2007), and concentration-response curves were obtained for GYY4137 and Na_2_S.

### Data and Statistical Analysis

All data were presented as mean ± S.E.M. with a significance level of *p* < 0.05, and n representing the number of individual animals (*n* > 5 for each protocol). Statistical comparisons between H2S release at time 0 and 10 h by GYY4137 and Na2S were performed by Student´s *t*-test. The two-way analysis of variance (ANOVA) was used to compare the different conditions affecting release of sulfide species from GYY4137 and concentration-response curves obtained in functional studies of isolated mesenteric arteries. The assumptions of the ANOVA approach were verified by inspection of Q-Q plots. The graphs and statistical analyses were performed using GraphPad Prism 7.0 (GraphPad Software, La Jolla, CA).

## Results

### Role of H_2_S in Na_2_S and GYY4137 Relaxation

**FIGURE 1:**
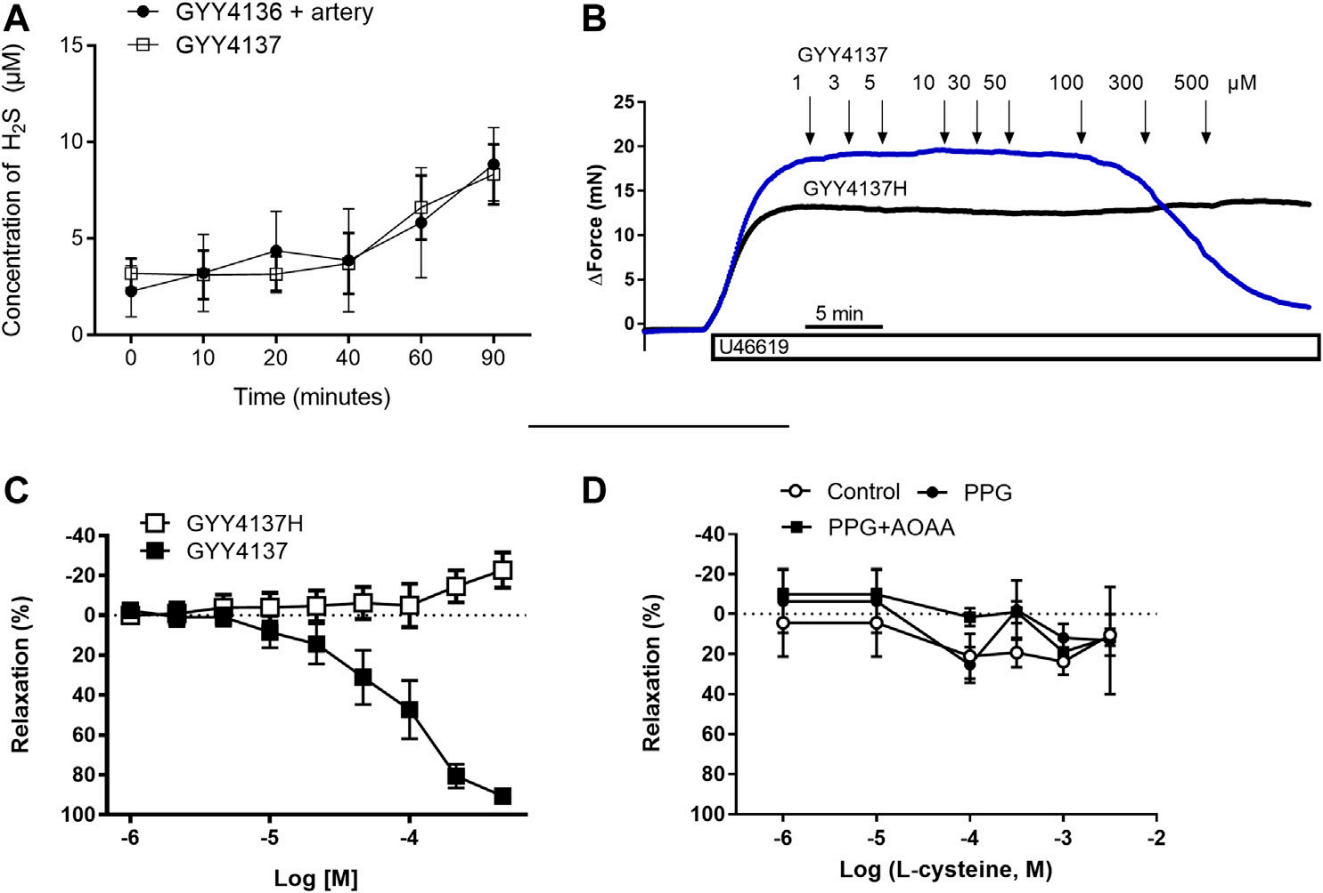
Role of H_2_S for GYY4137 relaxations small mesenteric arteries. **(A)** Sulfide release from GYY4137 in the absence and the presence of small mesenteric arteries **(B)** Original recordings in small mesenteric arteries showing contraction to 3 µM U46619 and addition of increasing concentrations of GYY4137 (1–500 µM) and the hydrolyzed form of GYY4137H (1–500 µM) **(C)** Average relaxations induced by the parent compound and the hydrolyzed form of GYY4137. **(D)** Effect of the substrate for H_2_S synthesis, L-cysteine, on vascular tone in small mesenteric arteries. The arteries were incubated with an inhibitors of H_2_S biosynthesis using the cystathionine-gamma-lyase (CSE) inhibitor, PPG (1 mM), and the cystathionine-beta-synthase (CBS)/cystathionine-gamma-lyase (CSE) inhibitor aminooxyacetic acid (AOAA, 1 mM). L-cysteine was added in increasing concentrations (1 µM-3 mM). Data are means ± SEM (*n* = 6).

The release of free H_2_S from GYY4137 was examined spectrophotometrically by the use of DTNB. Following previous studies (Li et al., 2008), incubation of 0.1 mM GYY4137 at pH 7.4 25°C resulted in a slow-release, which reached an end value of 8.33 µM after 90 min of incubation (Supplementary Figure S1A). This release was augmented by an increased starting concentration of GYY4137 (1 mM) (Supplementary Figure S1A). Sulfide release was also significantly increased under acidic conditions and by increased temperatures (Supplementary Figures S1B,C respectively). L-cysteine by itself increased the spectrophotometrically measured absorbance, but there was a further increase in sulfide release by combining cysteine and GYY4137 (Supplementary Figure S1D). The presence of rat mesenteric artery did not affect sulfide release from GYY4137 (Figure 1A). The results suggest that the release of sulfides from GYY4137 is independent of the presence of mesenteric artery.

Concentration-response curves were obtained in U46619 (0.3 µM)-contracted preparations for GYY4137 and the hydrolyzed product of GYY4137, GYY4137H to investigate whether the relaxant effects of GYY4137 were due to H_2_S release. We found that GYY4137 induced concentration-dependent relaxations while there was no change in vascular tone by adding the GYY4137H (Figures1B,C), suggesting that release of sulfides is pivotal for GYY4137 relaxation.

**FIGURE 2:**
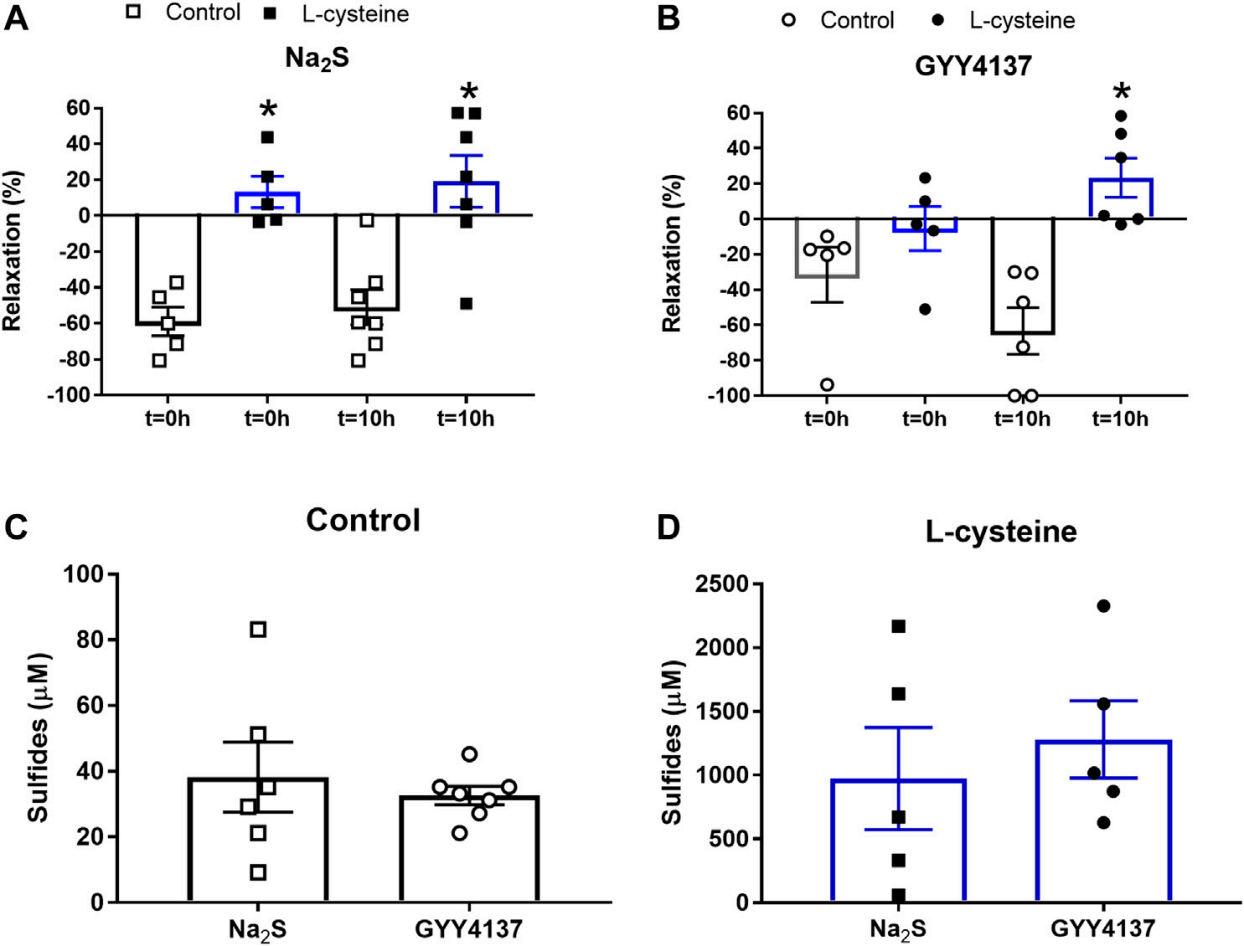
Effect of L-cysteine on Na_2_S and GYY4137 relaxations in rat small mesenteric arteries. Mixture of Na_2_S (300 µM) or GYY4137 (1 mM) with and without L-cysteine (1 mM) was added to air-tight bottles and relaxations measured to time = 0 and 10 h for **(A)** Na_2_S and **(B)** GYY4137, and the amount of sulfides measured at 10 h for **(C)** Na_2_S and GYY4137 in the absence, and for **(D)** Na_2_S and GYY4137 in the presence of L-cysteine, where the concentration of sulfides generated by L-cysteine were subtracted. Please note the difference in scale comparing data in **(C and D)**. Data are means ± SEM (*n* = 6). *p < 0.05, Students *t*-test.

**FIGURE 3:**
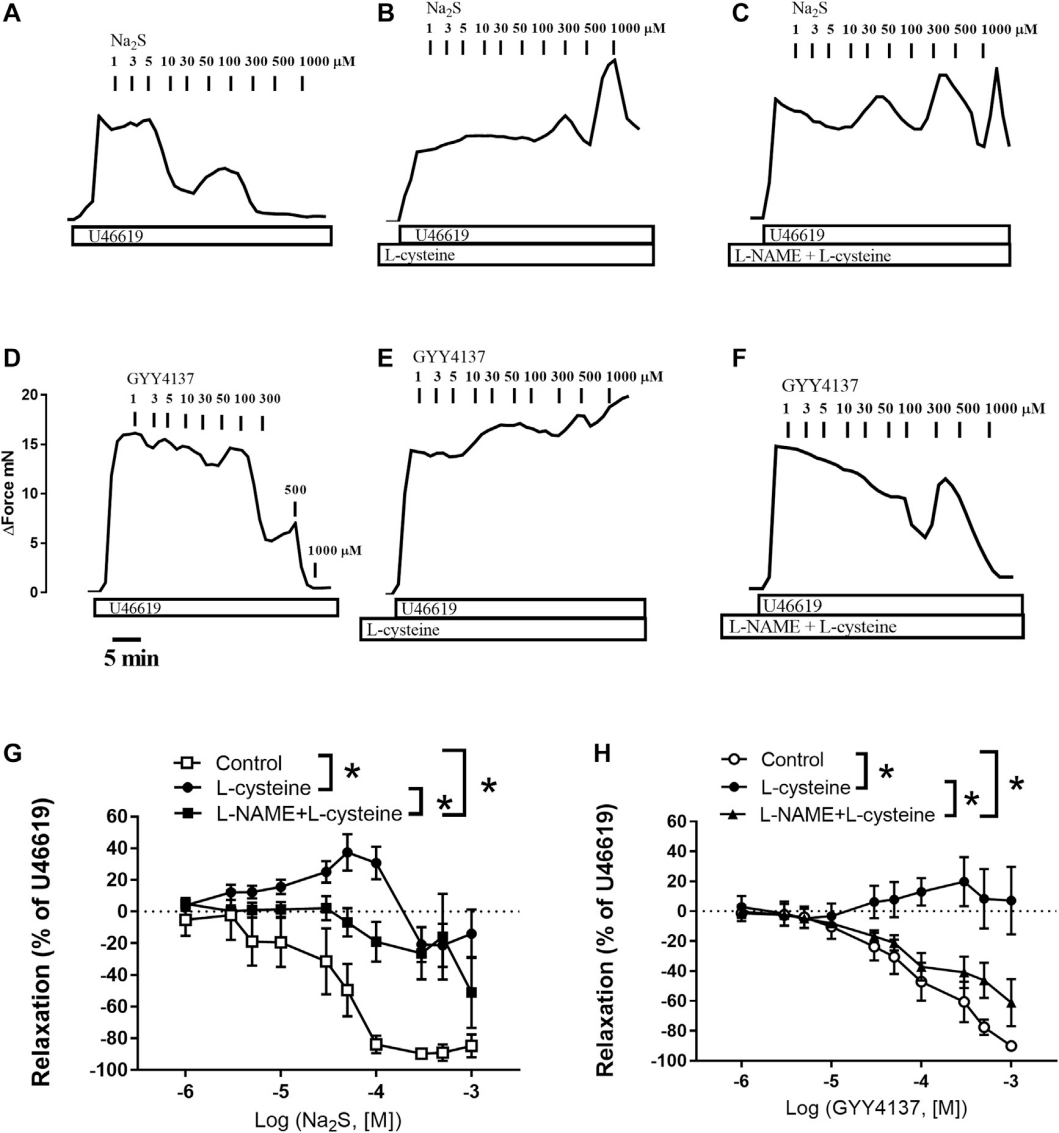
Effect of L-cysteine and L-NAME incubation on Na_2_S and GYY4137 relaxations in rat small mesenteric arteries. Original traces showing the effect of increasing concentrations of Na_2_S in U46619-contracted preparations in **(A)** control conditions **(B)** in the presence of L-cysteine (1 mM), and **(C)** in the presence of L-cysteine and the nitric oxide synthase inhibitor, L-NAME (100 µM). Original traces showing the effect of increasing concentrations of GYY4137 in U46619-contracted preparations in **(D)** control conditions **(E)** in the presence of L-cysteine (1 mM), and **(F)** in the presence of L-cysteine and the nitric oxide synthase inhibitor, L-NAME (100 µM). The horizontal bar indicates time in min and the vertical bar increase in force in mN **(G)** Average effect of L-cysteine without and with L-NAME on Na_2_S-induced vascular relaxation **(H)** Average effect of L-cysteine without and with L-NAME on GYY4137-induced vascular relaxation. Data are means ± SEM (*n* = 6). *p < 0.05, two-way ANOVA.

**FIGURE 4:**
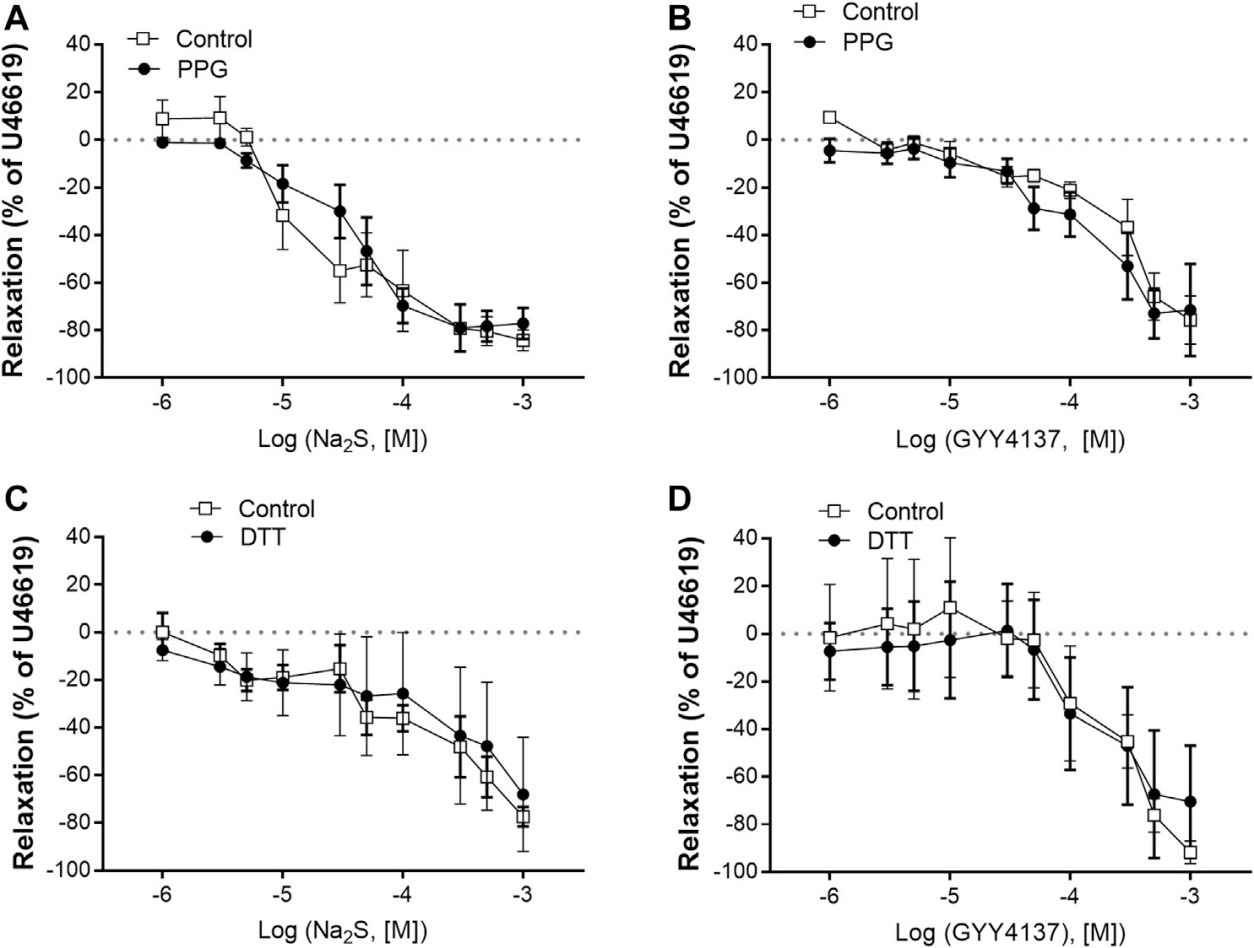
Effect of the cystathionine γ-lyase (CSE) inhibitor, PPG, and DTT on Na_2_S and GYY4137 relaxations in rat mesenteric arteries **(A)** Effect of PPG (1 mM) on Na_2_S induced vascular relaxation **(B)** effect of PPG on GYY4137 induced vascular relaxation **(C)** effect of DTT (1 mM) on Na_2_S induced vascular relaxation **(D)** effect of DTT on GYY4137 induced vascular relaxation. Where no error bars are indicated, error lies within dimensions of the symbol. *p < 0.05, two-way ANOVA. All data is represented as mean ± SEM (*n* = 5–6).

In U46619-contracted arteries with endothelium, the H_2_S substrate, and thiol-containing amino acid, L-cysteine (10^−6^–10^–2^ M) induced small relaxations which were 24 ± 6% at 10^–3^ M, and these relaxations were not inhibited in the presence of PPG (Figure 1D). Pre-mixing L-cysteine (10^–3^ M) with either Na_2_S or GYY4137 and adding it immediately after the mixing or 10 h later showed, that the presence of L-cysteine inhibited Na_2_S and GYY4137 relaxations (Figures 2A,B). The relaxations induced by Na_2_S and GYY4137 were comparable 10 h after the mixing of the solutions (Figures 2A,B), and this was also the case for the amount of sulfides measured in the solutions (Figures 2C,D). After preincubation with L-cysteine (10^−3^ M), instead of relaxations,3 × 10^–6^ to 10^–3^ M Na_2_S and 10^−4^–10^–3^ M GYY4137 induced contractions, which at the highest concentrations were contractions followed by relaxations (Figures 3A–D). In the presence of both L-NAME and L-cysteine, Na_2_S-induced contractions were reduced (Figures 3C,G), while the inhibitory effect of L-cysteine on GYY4137 relaxation was prevented (Figures 3F,H). To examine whether enzymatic conversion by CSE or interaction with thiol-groups play a role for Na_2_S and GYY4137 relaxations, the preparations were pre-incubated with an inhibitor of CSE, PPG (10^–3^ M), or the thiol reducing agent DTT (10^–3^ M), but these treatments did not change concentration-response curves for Na_2_S and GYY4137 (Figure 4).

Taken together our results show that L-cysteine converts Na_2_S and GYY4137 relaxations to contraction associated with markedly higher sulfide concentrations. This effect of L-cysteine on vascular tone was partly reversed in the presence of the endothelial NO synthase inhibitor, L-NAME.

### Effect of Endothelial Cell Removal and NO in Na_2_S and GYY4137 Relaxation

**FIGURE 5:**
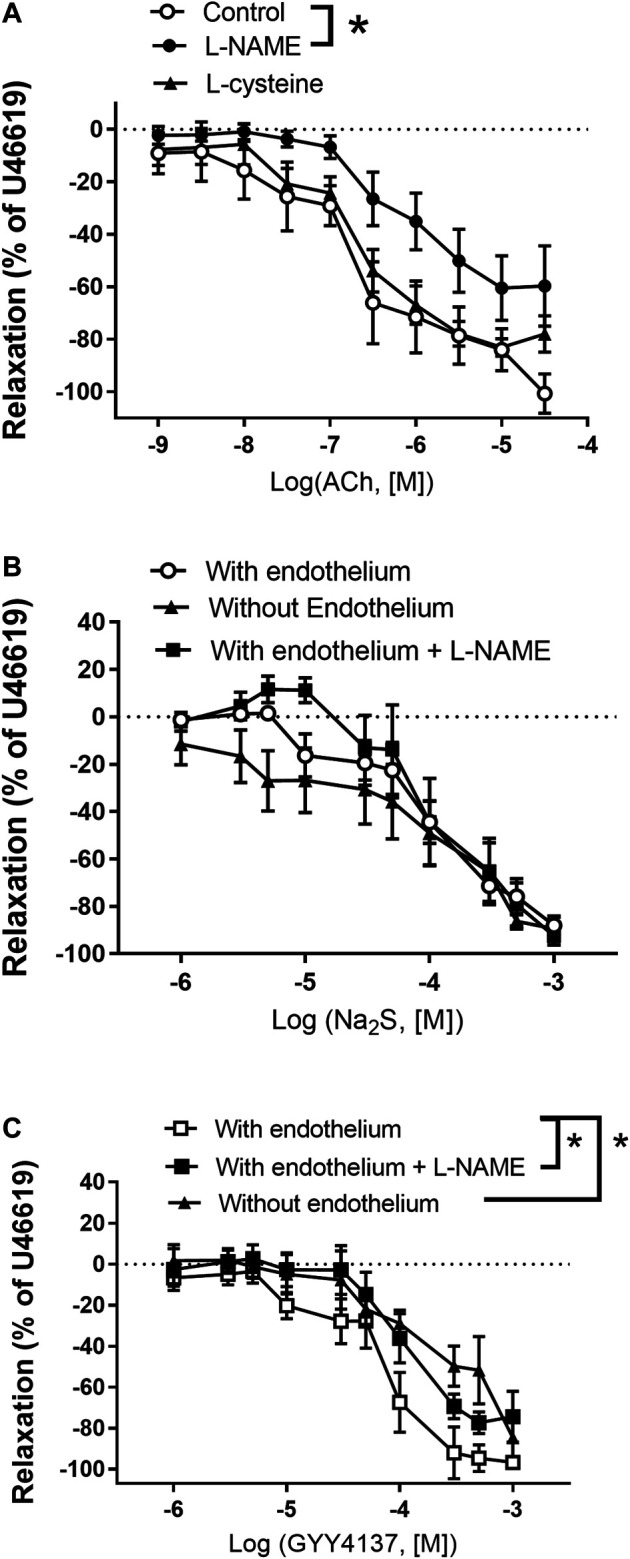
Role of the endothelium and NO for Na_2_S and GYY4137 relaxation **(A)** Average relaxations induced by acetylcholine (ACh) in the absence (*n* = 7) and the presence of either L-NAME (100 µM) (*n* = 6) or L-cysteine (1 mM) (*n* = 7) in rat mesenteric arteries **(B)** Na_2_S induced vascular relaxation in vessels with (*n* = 11) and without endothelium (*n* = 5), or with endothelium in the presence of an NO synthase inhibitor, L-NAME (*n* = 6) **(C)** GYY4137 induced vascular relaxation in vessels with (*n* = 12) and without endothelium (*n* = 6) or with endothelium in the presence L-NAME (*n* = 6). Where no error bars are indicated, error lies within dimensions of symbol. All data are means ± SEM. *p < 0.05, two-way ANOVA.

In contrast to L-cysteine (10^–3^ M), incubation with L-NAME significantly rightward shifted concentration-response curves for acetylcholine relaxation in small mesenteric arteries (Figure 5A). In U46619-contracted arteries, Na_2_S induced concentration-dependent relaxations, which were of similar magnitude in vessel segments with and without endothelium (Figure 5B), while concentration-response curves for GYY4137 were significantly rightward shifted in vessels without endothelium (Figure 5C). In the presence of an inhibitor of NO synthase, L-NAME (10^–4^ M), the concentration-response curves for Na_2_S were unaltered (Figure 5B), while L-NAME rightward shifted concentration-response curves for GYY4137 (Figure 5C). These results suggest that in rat small mesenteric arteries, endothelium-derived NO is of importance for some of the effects of GYY4137 on vascular tone, while there were no significant differences for Na_2_S.

**FIGURE 6:**
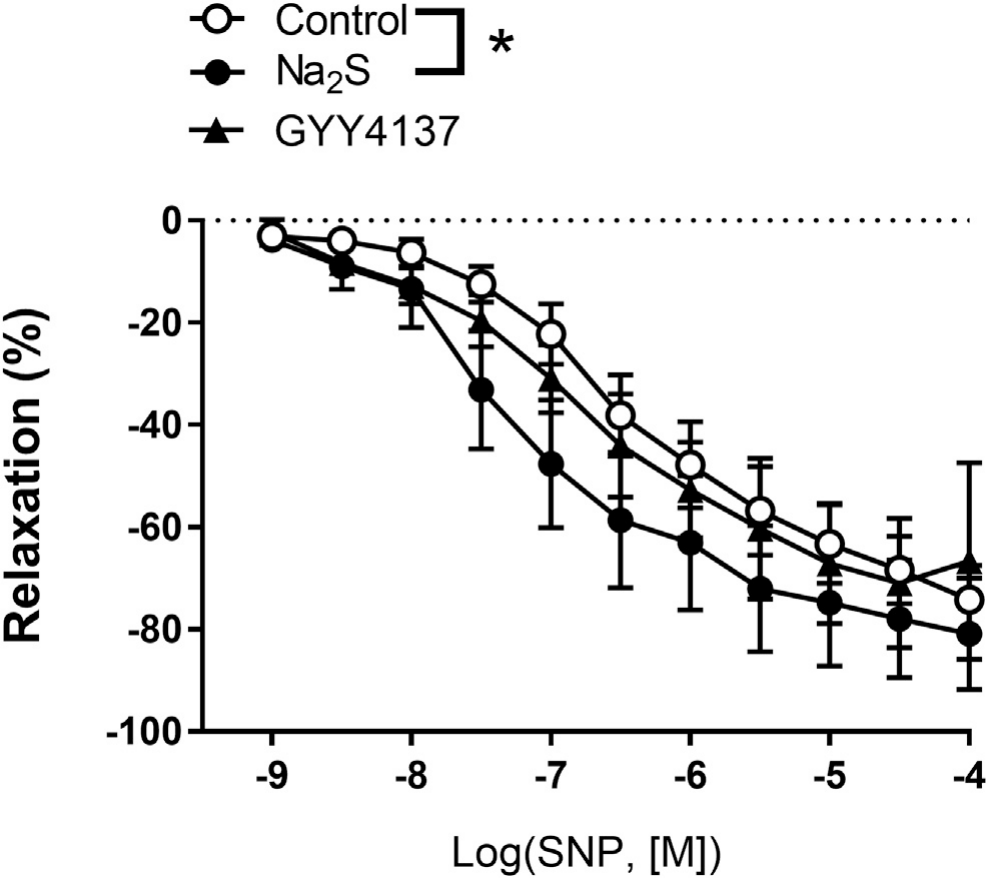
Effect of Na_2_S and GYY4137 on relaxations induced by the NO donor, sodium nitroprusside (SNP) in small mesenteric arteries. The arteries were incubated with and inhibitor of NO synthase, L-NAME (10 µM), and Na_2_S (300 µM) (*n* = 6), or GYY4137 (1 mM) (*n* = 5), contracted with U46619 (0.3 µM) and concentration-response curves constructed for SNP. *p < 0.05, two-way ANOVA. All data are means ± SEM.

To investigate the effect of Na_2_S and GYY4137 on NO donor-induced relaxations, the small mesenteric arteries were incubated with vehicle, Na_2_S (3 × 10^–4^ M), or GYY4137 (10^–3^ M), then contracted to the same level with U46619 (0.3 µM), and increasing concentrations of SNP was added. We found that in the presence of Na_2_S, concentration-response curves for SNP were leftward shifted, while the presence of GYY4137 did not change the relaxation responses for SNP in small mesenteric arteries (Figure 6).

### Involvement of K Channels in GYY4137 and Na_2_S-Induced Vascular Relaxation

**FIGURE 7:**
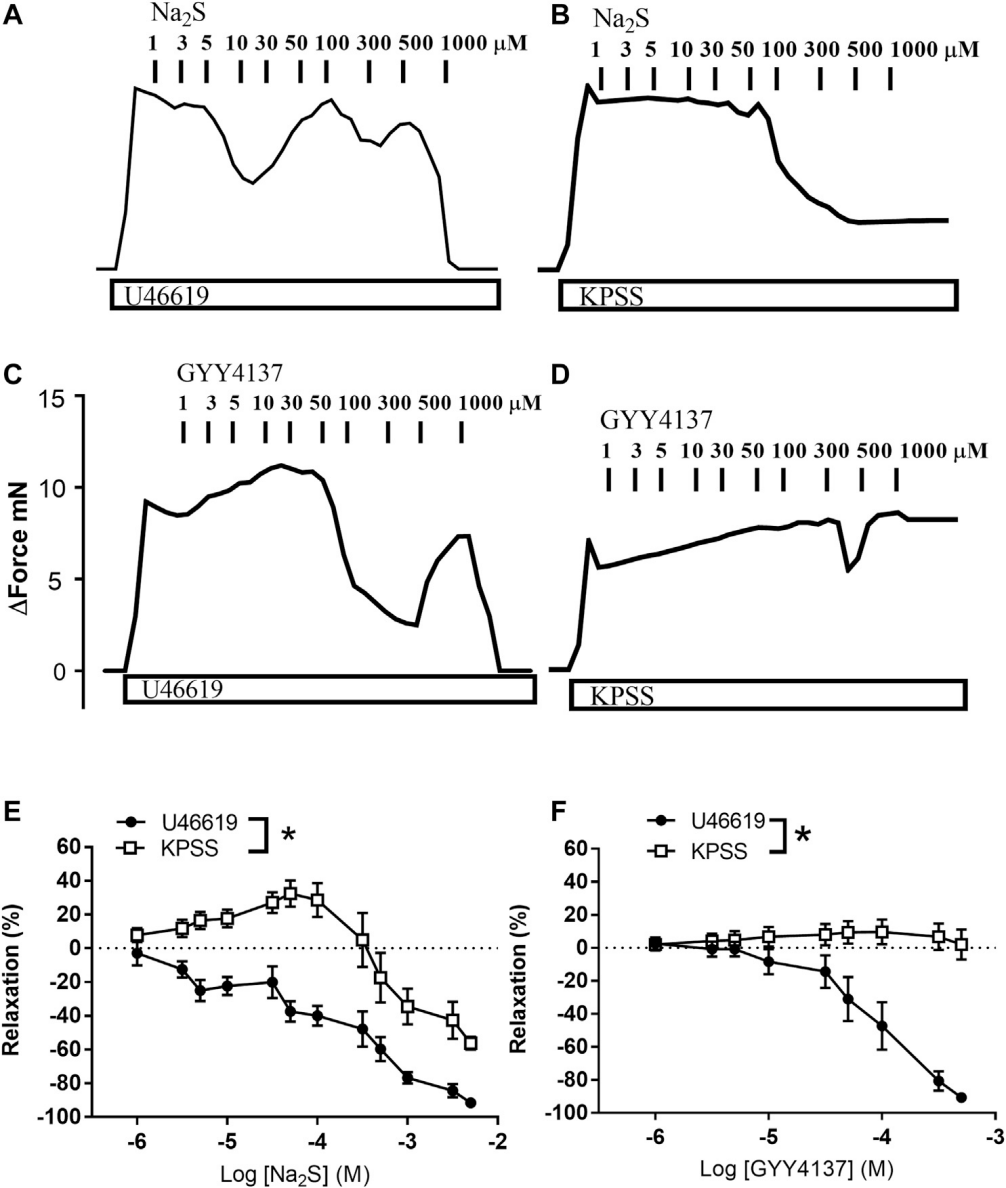
Effect of high extracellular potassium (KPSS) on Na_2_S and GYY4137 relaxation. Original traces showing contractions induced by 3 µM U46619 or 60 mM KPSS and effect of increasing concentrations of **(A,B)** Na_2_S (1–1,000 µM) or **(C,D)** GYY4137 (1–1,000 µM). **(E)** Average Na_2_S induced vascular relaxations in vessels contracted with U46619 or KPSS; **(F)** Average GYY4137 induced vascular relaxations in vessels contracted with U46619 or KPSS. Where no error bars are indicated, error lies within dimensions of symbol. All data are means ± SEM (*n* = 5–10). *p < 0.05, two-way ANOVA.

**FIGURE 8:**
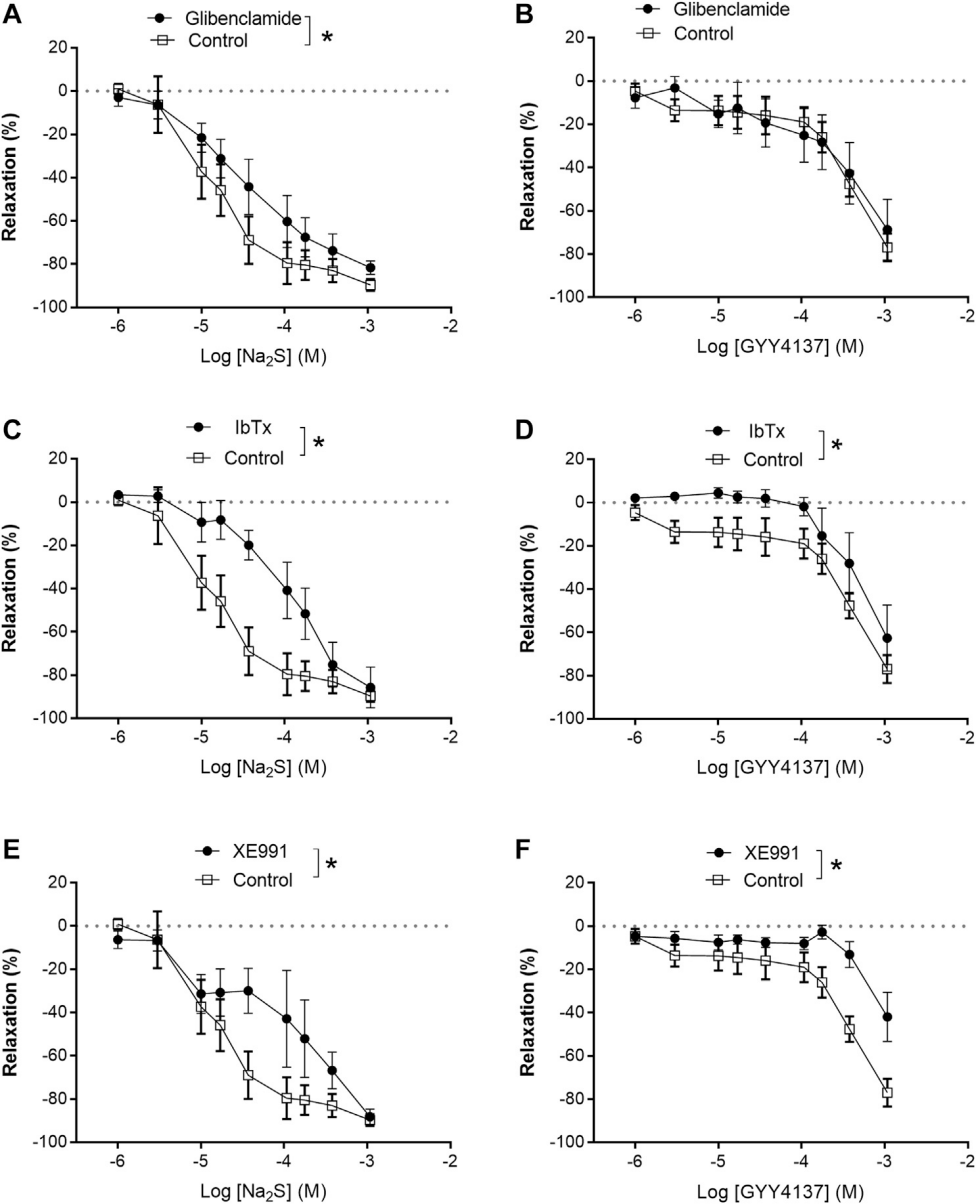
Effect of K channels blockers on Na_2_S and GYY4137 relaxation. **(A)** Effect of inhibition of K_ATP_ channels by glibenclamide (1 µM) on Na_2_S-induced vascular relaxation; **(B)** effect of inhibition of K_ATP_ channels by glibenclamide on GYY4137-induced vascular relaxation; **(C)** effect of selective blockade of BK_Ca_ channels by iberiotoxin (IBTX, 100 nM) on Na_2_S-induced vascular relaxation; **(D)** effect of selective blockade of BK_Ca_ channels by IBTX on GYY 4137-induced vascular relaxation; **(E)** effect of selective blockade of Kv7 channels by XE991 (10 µM) on Na_2_S-induced vascular relaxation; **(F)** effect of selective blockade of K_V_7 by XE991 on GYY4137-induced vascular relaxation. Where no error bars are indicated, errors lies within dimensions of symbol. All data are means ± SEM (*n* = 8–10). *p < 0.05, two-way ANOVA.

In preparations contracted with high extracellular potassium (60 mM KPSS), relaxations induced by GYY4137 were abolished, while relaxations induced by Na_2_S were significantly decreased compared with responses obtained in U46619 (0.3 µM)-contracted preparations (Figures 7A–D). In contrast to GYY4137, Na_2_S still induced 60% maximum relaxation in 60 mM KPSS-contracted preparations (Figures 7E,F), hence suggesting that K channels are pivotal for GYY4137-induced relaxations, while K channels and also other mechanisms contribute to Na_2_S relaxation. To investigate the K channel subtypes involved in the relaxations, the preparations were incubated with blockers of ATP-sensitive K channels (glibenclamide), BK_Ca_ (iberiotoxin), and of K_V_7 channels (XE991). Glibenclamide decreased Na_2_S relaxation, while GYY4137 relaxation was unaltered in U46619-contracted arteries (Figures 8A,B). Iberiotoxin and XE991 significantly decreased relaxations induced by both Na_2_S and GYY4137 (Figures 8C–F).

## Discussion

The main findings in the present study are that GYY4137 spontaneously releases low amounts of sulfides leading to relaxation, and that L-cysteine by direct chemical interaction inhibits Na_2_S and GYY4137 relaxations. The observation of sulfide release is supported by the tissue-independent effect on sulfide release measured from GYY4137 and that the hydrolyzed control, GYY4137H, in contrast to the parent molecule, fails to relax small mesenteric arteries. Moreover, Na_2_S induced comparable relaxations after dissolving at 0 h compared to 10 h storage, while GYY4137 relaxation was markedly increased by storage for 10 h in airtight containers and yielded sulfide accumulation similar to Na_2_S, and suggesting GYY4137 is associated with slow release of H_2_S. These findings suggest rate of H_2_S release plays an essential role for the effect on vascular tone, where high levels of H_2_S from Na_2_S interacts leftward shift concentration-response curves for the NO donor SNP, while low levels of H_2_S from GYY4137 interact with endogenous endothelium-derived NO leading to relaxation. Moreover, blockers of K_ATP_, BK_Ca_, and K_V_7 channels affected Na_2_S and GYY4137 concentration-response curves differently.

It has previously been shown that GYY4137 releases H_2_S (Li et al., 2008; Whiteman et al., 2010; Martelli et al., 2013b), and in agreement with these studies, we found that GYY4137 concentration-dependently releases small amounts of sulfides. This release is markedly enhanced by increasing the temperature and acidifying the solutions. In contrast, at physiological conditions (pH 7.4, 37°C), we found by simultaneous measurements of H_2_S gas and relaxation that GYY4137 caused relaxation of small rat arteries without releasing detectable amounts of H_2_S gas (Hedegaard et al., 2016). However, the lack of relaxant effect of the hydrolyzed GYY4137 control compound, GYY4137H (Alexander et al., 2015), suggests that GYY4137-induced vessel relaxation requires H_2_S.

Several H_2_S releasing compounds with slow releasing rates, including organic polysulphides of garlic, e.g., diallyl disulfide and arylthiamides require the presence of reduced glutathione or thiols to release H_2_S (Benavides et al., 2007; Martelli et al., 2013b). However, in the presence of arterial tissue, the amount of sulfides measured from GYY4137 was not increased suggesting the H_2_S release is tissue-independent. Moreover, Na_2_S induced comparable relaxations immediately after dissolving the salt compared to solutions stored in airtight containers for 10 h, while GYY4137 stored for 10 h yielded sulfide accumulations similar to Na_2_S and markedly increased relaxation. These findings suggest rate of H_2_S release plays an essential role for the effect on vascular tone of GYY4137.

Plasma L-cysteine concentrations are in the range of 3.5–11 μmol/L (Chawla et al., 1984) and L-cysteine is considered one of the primary substrates leading to formation of H_2_S. It has at high concentrations been found to increase formation of H_2_S in mammalian tissues such as kidney (Jackson-Weaver et al., 2013), and to cause relaxations in small cerebral (Streeter et al., 2012) and retinal arteries (Takır et al., 2015), although 1–300 μM L-cysteine had no effect (Takır et al., 2015). In agreement with the latter study, we only observed small relaxations by adding increasing concentrations of L-cysteine to U46619-contracted arteries and no effect on acetylcholine relaxation. In rat mesenteric arteries, the expression of CSE is high in perivascular and adventitial tissue and associated with formation of H_2_S (Jackson-Weaver et al., 2011; Li et al., 2013). In the present study, we carefully removed adhering tissue and cannot exclude L-cysteine will contribute to endogenous H_2_S formation to a larger degree in mesenteric arteries with adhering perivascular tissue.

L-cysteine is considered a scavenger of nitroxyl (HNO) (Andrews et al., 2009), and studies in cell cultures reported that L-cysteine by direct interaction may scavenge HS^−^ and lead to formation of inactive sulfides (Miyamoto et al., 2017). By mixing L-cysteine with Na_2_S or GYY4137, we observed increased accumulation of sulfides (Figure 2). Considering that L-cysteine by itself only had small effect on vascular tone and did not change acetylcholine relaxation, the effect of L-cysteine on Na_2_S and GYY4137 may be ascribed to a direct chemical reaction, and thereby inactivating Na_2_S and GYY4137 relaxation.

**FIGURE 9:**
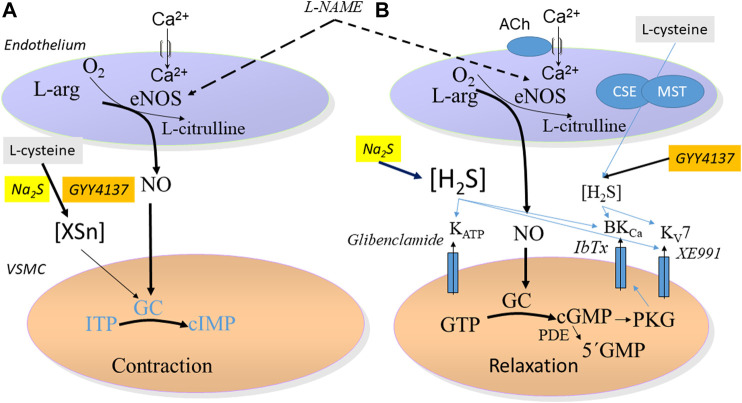
Schematic figure proposing the mechanisms involved in **(A)** contraction in small mesenteric arteries induced by Na_2_S and GYY4137 in the presence of L-cysteine. This involve a formation of a sulfide product (XSn) that may modulate guanylate cyclase leading to contraction of the vascular smooth muscle cells (VSMC). The effect is partly reversed by the NO synthase inhibitor, L-NAME **(B)** Relaxation induced by Na_2_S and GYY4137 leads to, respectively, fast and slow release of H_2_S and followed by activation of subsets of different potassium channels: ATP-sensitive K (K_ATP_) channels by Na_2_S, and of large-conductance calcium activated (BKCa) and voltage-gated type 7 (K_V_7) channels by GYY4137.

In previous studies, NaSH and Na_2_S induced contraction followed by relaxation is observed in the perfused mesenteric vascular bed and in isolated arteries (Ali et al., 2006; Di Villa Bianca et al., 2011; Hedegaard et al., 2016). In the presence of L-cysteine, low concentrations of Na_2_S and GYY4137 induced marked contractions of the isolated rat mesenteric arteries, and when L-cysteine was mixed with Na_2_S or GYY4137, we observed an increased sulfide accumulation. Polysulfides (H_2_S_2_ and H_2_S_3_) have been suggested to play a role in the effects of H_2_S or to produce many of the effects previously attributed to H_2_S (Kimura, 2019), but the effects of these unstable compounds were reported to be inhibited in the presence of L-cysteine (Miyamoto et al., 2017), and in previous studies we observed that polysulfides (K_2_S_n_) induce relaxations in rat mesenteric arteries (Hedegaard et al., 2016). Therefore, it seems unlikely that L-cysteine by interaction with Na_2_S and GYY4137 just leads to formation of inactive sulfides. Instead the conversion of the Na_2_S and GYY4137 relaxations to contractions by L-cysteine treatment may result from inhibition of polysulfides. However, inhibition of formation of polysulfides with DTT (Miyamoto et al., 2017) did not affect Na_2_S and GYY4137 relaxation (Figures 4C,D). Therefore, a more speculative mechanism is that L-cysteine together with Na_2_S or GYY4137 result in the formation of a product that may interfere with endothelial NO synthase leading to contraction, e.g., formation of cIMP instead of cGMP by NO as described in large coronary arteries (Chen et al., 2014) (Figure 9A). Indeed in the presence of L-cysteine, L-NAME by inhibition of NO synthase restored the GYY4137 relaxations. In agreement with these findings we in preliminary studies observed in ^1^H-, ^31^P-NMR spectra that L-cysteine incubation with GYY4137 formed a product (results not shown) which support the formation of a product that may interfere with vascular tone in small mesenteric arteries, although other experimental approaches will be required to characterize the product formed by L-cysteine and GYY4137.

While 500 and 1,000 nmol/kg Na_2_S failed to change blood pressure in normotensive rats (Tomasova et al., 2015), intravenous injection of 39 μmol/kg Na_2_S decreased mean arterial blood pressure with 45 mmHg in anesthetized mice (Eberhardt et al., 2014). Treatment with 56 μmol/kg/day of NaSH administered by intraperitoneal injection lowered also blood pressure in spontaneously hypertensive rats (Ni et al., 2018). These findings suggest that high doses of H_2_S salts lowers blood pressure by vasodilatation. As mentioned in the introduction, several mechanisms have been suggested to mediate NaSH and Na_2_S vasodilatation, depending on the vascular preparations that have been studied. In previous studies in resistance arteries, H_2_S vasodilatation was found to involve K_ATP_ channels (Tang et al., 2005), BK_Ca_ channels (Jackson-Weaver et al., 2011; Jackson-Weaver et al., 2013), and K_V_7 channels (Schleifenbaum et al., 2010; Hedegaard et al., 2016). In patch-clamp studies H_2_S gas 30 μM to 1 mM caused activation of K_ATP_ channels in vascular smooth muscle from mesenteric arteries (Tang et al., 2005), and 10 µM NaSH hyperpolarized mesenteric arteries by iberiotoxin-sensitive mechanism also suggesting the involvement BK_Ca_ channels (Jackson-Weaver et al., 2011, 2013), and NaHS (1 mM) hyperpolarized rat aorta and directly activated K_V_7 channels in CHO cells (Martelli et al., 2013a). Recent studies have also shown that direct activation of K_V_7 channels by H_2_S donors protects against neuropathic pain (Di Cesare Mannelli et al., 2017), and that direct persulfidation of K_V_7 channels by H_2_S plays an important role in skeletal muscle hypercontractility in human malignant hyperthermia syndrome (Vellecco et al., 2020). In agreement with studies in resistance arteries, in small mesenteric arteries contracted with the U46619, Na_2_S in the present study induced relaxations sensitive to high extracellular potassium and blockers of both ATP-sensitive, K_V_7, and BK_Ca_ channels suggesting involvement of these channels in Na_2_S relaxation, although electrophysiological measurements, e.g., membrane potential measurements or patch-clamp will be required to confirm the activation of K_V_7 channels by Na_2_S in this preparation.

Interaction of H_2_S with the NO pathway is thought to be important for the vascular effects of Na_2_S. Thus, it was proposed that NO and H_2_S may act co-operatively to generate nitroxyl (HNO), and that this activates transient receptor potential ankyrin 1 (TRPA1) channels on sensory nerves with subsequent calcitonin gene-related peptide release and relaxation in meningeal and mouse mesenteric arteries (Eberhardt et al., 2014). In constrast, based on studies in mice with downregulation of CSE, it was suggested that physiological concentrations of H_2_S scavenge endothelium-derived NO, and in the absence of NO leads to activation of smooth muscle K_ATP_ and K_V_ channels (Szijártó et al., 2018). In the present study, endothelial cell removal or inhibition of NO synthase failed to change relaxations induced by Na_2_S in mesenteric arteries. These findings agree with our previous studies showing that NaSH relaxation in rat mesenteric arteries is NO and endothelium-independent. However, incubating the preparations with Na_2_S leftward shifted concentration-response curves for an exogenous NO donor, SNP suggesting that high concentrations of Na_2_S and NO synergistically cause vasodilatation in rat mesenteric arteries. Moreover, our results support that Na_2_S causes relaxation by activation of K channels in the smooth muscle layer. High concentrations of Na_2_S also relax contractions induced by high extracellular potassium suggest that K channel independent mechanisms are involved (Figure 5A), and may similar to NaSH involve inhibition of mitochondrial complex I and III (Hedegaard et al., 2016).

The mechanism of GYY4137 induced vascular relaxation has previously been observed to be endothelium-dependent in rat aorta rings (Li et al., 2008). In small mesenteric arteries, GYY4137 relaxations were reduced in preparations without endothelium, and by an inhibitor of NO synthase suggesting endothelium-derived NO is involved in relaxations induced by GYY4137. Interestingly, incubation with GYY4137 failed to change relaxations induced by SNP suggesting that high H_2_S concentrations are required to act synergistically with an NO donor, but also implying that the interaction of GYY4137 with endothelium-derived NO is likely at smooth muscle level.

In aorta segments and ciliary arteries K_ATP_ channels were found involved in GYY4137 relaxation (Li et al., 2008; Chitnis et al., 2013). Here, we provide evidence that potassium channels may play a pivotal role in the vascular relaxations induced by GYY4137, as high extracellular potassium completely inhibited GYY4137 relaxation. Also, blockers of smooth muscle K_V_7 and BK_Ca_ channels, XE991 and iberiotoxin markedly inhibited relaxation, suggesting these channels are involved in relaxations induced by GYY4137 in rat mesenteric arteries (Figure 9B). Therefore, the mechanisms involved in GYY4137 relaxation regarding both the endothelium and involvement of K channels seems different from the mechanisms involved in Na_2_S relaxation, reflecting different rate and levels of H_2_S reaching the vascular preparations when Na_2_S salt and GYY4137 are added to an organ bath in similar conditions.

## Conclusion and Perspectives

The present findings suggest that L-cysteine by reaction with Na_2_S and GYY4137 and formation of sulfides, inhibits relaxations by these compounds. The low rate of release of H_2_S species from GYY4137 is reflected by the different sensitivity of these relaxations towards high K^+^ concentration and K channel blockers compared with Na_2_S. The perspective is that the rate of release of sulfides plays an important for the effects of H_2_S salt vs. donors in small arteries.

## Data Availability

The raw data supporting the conclusions of this article will be made available by the authors, without undue reservation.
