# Supplementary material for: GYY4137 and Sodium Hydrogen Sulfide Relaxations Are Inhibited by L-Cysteine and KV7 Channel Blockers in Rat Small Mesenteric Arteries
Source: Front Pharmacol. 2021 Mar 26;12:613989. doi: 10.3389/fphar.2021.613989 (PMC8032876; doi:10.3389/fphar.2021.613989)

GYY4137 and sodium hydrogen sulfide relaxations are inhibited by L-cysteine and K_V7 channel blockers in rat small mesenteric arteries

Silvijus Abramavicius[1,2], Asbjørn G. Petersen[1], Nirthika S. Renaltan[1], Judit Prat-Duran[1], Roberta Torregrossa[3], Edgaras Stankevicius[2], Matthew Whiteman[3], Ulf Simonsen[1]*

Supplementary Figure for review. ^{1}H-, ^{31}P-NMR spectra five hours after mixing GYY4137 mixed with five equivalents of L-cysteine in water. The blue arrows show the formation of a new product. The results are preliminary and number of other approaches will be required to identify the product that is formed.

^{1}H-, ^{31}P-NMR spectra relative to GYY4137 with 5 eq. of cysteine in water after 5 h:

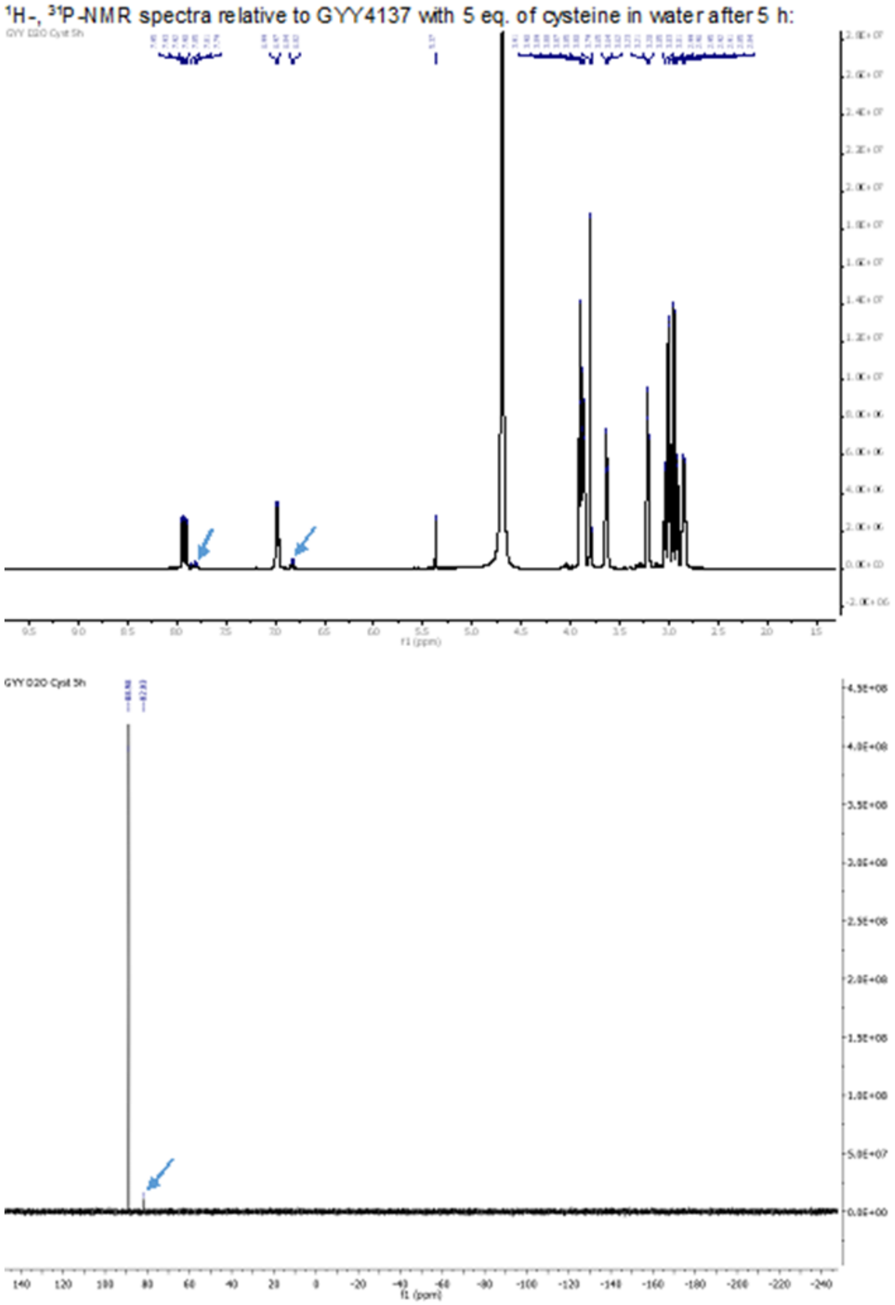

Supplement: Supplementary file 1 [file datasheet1.pdf]
