# Supplementary material for: GYY4137 and Sodium Hydrogen Sulfide Relaxations Are Inhibited by L-Cysteine and KV7 Channel Blockers in Rat Small Mesenteric Arteries
Source: Front Pharmacol. 2021 Mar 26;12:613989. doi: 10.3389/fphar.2021.613989 (PMC8032876; doi:10.3389/fphar.2021.613989)
Supplement: Supplementary file 2 [file datasheet2.pdf]

Supplementary Material

GYY4137 and sodium hydrogen sulfide relaxations are inhibited by L-cysteine and K_V7 channel blockers in rat small mesenteric arteries

Silvijus Abramavicius[1,2], Asbjørn G. Petersen[1], Nirthika S. Renaltan[1], Judit Prat-Duran[1], Roberta Torregrossa[3], Edgaras Stankevicius[2], Matthew Whiteman[3], Ulf Simonsen[1]*

[1]Department of Biomedicine, Pulmonary and Cardiovascular Pharmacology, Aarhus University, Aarhus, Denmark
[2]Institute of Physiology and Pharmacology, Lithuanian University of Health Sciences, Kaunas, Lithuania
[3]University of Exeter Medical School, St. Luke's Campus, Exeter, United Kingdom

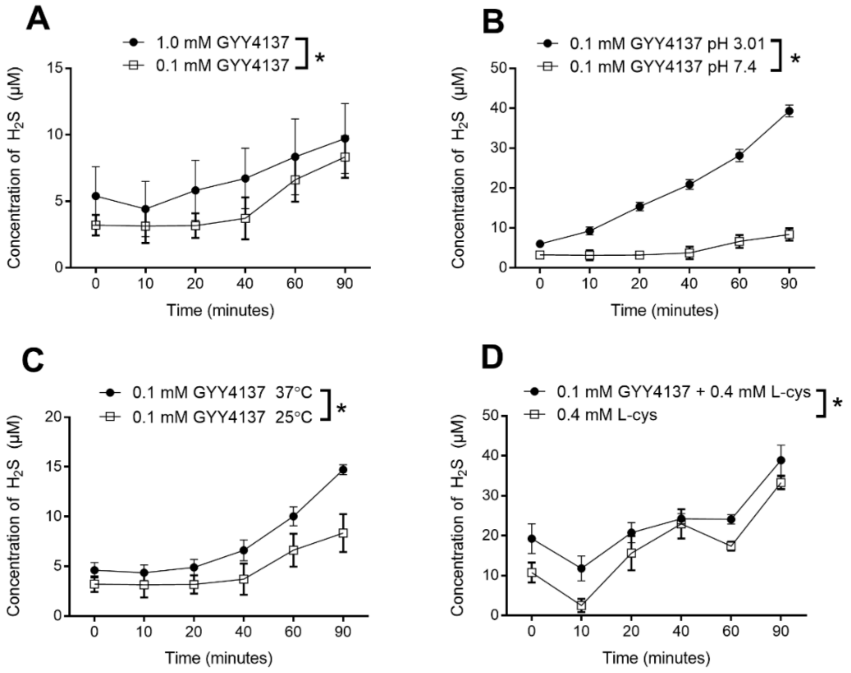

Supplementary Figure S1. Release of sulfides from GYY4137 under different conditions. A) Effect of GYY4137 concentration. B) Effect of pH. C) Effect of temperature. D) Effect of L-Cysteine on release of sulfides. The increase in sulfides caused by L-cysteine were subtracted and the figure only show the increase induced after addition of GYY4137. Where no error bars are indicated, error lies within dimensions of symbol. *$P<0.05$, two-way ANOVA. All data are represented as means ± SEM (n = 6).
